# Intraoperative triggered electromyographic monitoring of pedicle screw efficiently reduces the lumbar pedicle breach and re-operative rate-a retrospective analysis based on postoperative computed tomography scan

**DOI:** 10.1186/s12891-023-06658-6

**Published:** 2023-06-30

**Authors:** Tong Yongjun, Zhao Yuntian, Chen Biao, Jiang Zenghui

**Affiliations:** 1 Department of Orthopedics, Zhejiang Hospital, No.1229, Gudun Rd, Hangzhou, 310030 Zhejiang China; 2 Sage Ridge School, 2515 Crossbow Ct, Reno, NV 89511 USA

**Keywords:** Triggered electromyography, Pedicle screw breach, Revision, Screw-nerve root distance

## Abstract

**Background:**

To investigate whether intraoperative triggered electromyographic (T-EMG) monitoring could effectively reduce the breach rate of pedicle screws and the revision rate.

**Methods:**

Patients with posterior pedicle screw fixation from L1-S1 were enrolled between June 2015 and May 2021. The patients in whom T-EMG was utilized were placed in the T-EMG group, and the remaining patients were considered in the non-T-EMG group. Three spine surgeons evaluated the images. The two groups were divided into subgroups based on screw position (lateral/superior and medial/inferior) and breach degree (minor and major). Patient demographics, screw positions, and revision procedures were reviewed.

**Results:**

A total of 713 patients (3403 screws) who underwent postoperative computed tomography (CT) scans were included. Intraobserver and interobserver reliabilities were perfect. The T-EMG and non-T-EMG groups had 374(1723 screws) and 339 (1680 screws) cases, respectively. T-EMG monitoring efficiently reduced the overall screw breach (T-EMG 7.78% vs. non-T-EMG 11.25%, p = 0.001). in the subgroup analysis, the medial/inferior breach rate was higher in the T-EMG group than in the non-T-EMG group (T-EMG 6.27% vs. non-T-EMG 8.93%, p = 0.002); however, no difference was observed between the lateral and superior breaches (p = 0.064). A significant difference was observed between the minor (T-EMG 6.21% vs. non-T-EMG 8.33%, p = 0.001) and major (T-EMG 0.06% vs. non-T-EMG 0.6%, p = 0.001) medial or inferior screw breach rates. Six screws (all in the non-T-EMG group) underwent revision, with a significant difference between the groups (T-EMG 0.0% vs. non-T-EMG 3.17%, p = 0.044).

**Conclusions:**

T-EMG is a valuable tool in improving the accuracy of screw placement and reducing the screw revision rate. The screw-nerve root distance is vital in causing symptomatic screw breach.

**Trial registration:**

The study is retrospective registered in China National Medical Research Registration and Archival information system in Nov 17th 2022.

## Background

Low back pain is highly prevalent, and the main cause of years lived with disability in the adult population with an estimated lifetime prevalence of 70–85% [1, 2]. From 1990 to 2019, the incidence of low back pain increased by 50% [3]. Although only 1.2% of those patients receive a surgical intervention [4], the use of spinal surgery has increased dramatically over the last decades [5, 6], from 60.4 to 100,000 in 2004 to 79.8 per 100,000 in 2015 in united states [7]. Posterior lumbar pedicle screw fixation is one of the most widely used techniques in spinal surgery. It is often used for spondylolisthesis, spine fractures, scoliosis, and spondylodiscitis, where pedicle screw fixation can provide good stability to the spine [8].

However, pedicle screw fixation is not without risks. The literature reported that the pedicle screw breach rate ranges from 5.5–40% [9, 10]. Most patients with pedicle screw breaches have no significant symptoms [11], while severe screw breaches of the medial and inferior pedicle walls can cause catastrophic consequences. Further, reoperation is required for patients with symptomatic screw breaches, making it essential to reduce the pedicle screw breach rate [12, 13].

Previous methods to decrease the pedicle screw breach rate include pedicle ball-tip probes and intraoperative X-ray fluoroscopy, but these methods have drawbacks. The reports found that the pedicle ball-tip probe was not suitable as an assessment tool alone, as it had a false negative effect when detecting the pedicle wall [14]. Moreover, under similar conditions, intraoperative fluoroscopy was not always accurate in assessing medial wall violation of pedicle [15].

Furthermore, literatures reported several new methods to reduce the screw breach rate. The use of intraoperative O-arm navigation or intraoperative computed tomography (CT), a novel technique with increasing utility in recent years that is expensive and radiologically hazardous [16, 17], remains controversial [18, 19]. Three-dimensional (3D) printed screw guide templates have been used in spinal deformity surgery for years [20]. Though relatively accurate, screw trajectory deviation has been reported to occur up to 17% with use of 3D printed guides, probably due to a poor fit between template and bone [21]. The robot-assisted pedicle screw placement is prevalent during the last few years and has the potential to increase the accuracy while decreasing the radiated exposure, complication rate [22]. However, robot requires high costs and long-learning curve [22]. The probe with electronic conductivity device significantly reduced the incidence of misplaced screw which is highly relied on instruments [23, 24].

Intraoperative neurophysiological monitoring, which is widely used in spinal surgery, plays an important role in reducing intraoperative nerve injury [14, 25]. Triggered electromyography (T-EMG) has been reported as a method for evaluating the accuracy of intraoperative screw placement [26, 27]. The intraoperative EMG monitoring system was not available in our hospital until April 1st, 2018. Since then, intraoperative EMG monitoring becomes a routine procedure during spine surgery in our hospital. However, the conclusions of the articles on whether T-EMG could effectively reduce the screw breach rate are ambiguous [28–33].

We retrospectively analyzed the data on posterior lumbar pedicle screw position with (after April 1st, 2018) or without (before April 1st, 2018) intraoperative T-EMG monitoring based on postoperative CT in our hospital to investigate whether intraoperative T-EMG monitoring could effectively reduce the breach rate of pedicle screws and the revision rate of screw breaches.

## Materials and methods

### Ethical approval

This study was conducted in accordance with the Declaration of Helsinki and was approved by the ethical committee of our university hospital. As the current study was retrospective in nature and data were analyzed anonymously, this study was exempt from requiring informed consent from patients.

### Patient demographics

We retrospectively identified patients who underwent posterior pedicle screw fixation at our hospitals between June 2015 and May 2021. These patients were operated on by a senior attending surgeon. The inclusion criteria were as follows: (1) indications for spinal surgery were spinal fracture, lumbar spinal stenosis, lumbar disc herniation, spondylolisthesis, scoliosis, tumor, and spondylodiscitis; (2) pedicle screws were placed from L1-S1; (3) immediate postoperative CT scan of the operative lumbar spine.

The exclusion criteria were as follow: (1) preoperative CT scan without an intact pedicle wall, (2) congenital dysmorphic pedicle features, (3) hollow pedicle screws, (4) patients who underwent minimally invasive surgery with percutaneous screw insertion, and (5) the vertebras for which the surgeons determined not to reinsert pedicle screws after t-EMG ≤ 15 mA.

A total of 713 patients with posterior pedicle screw fixation at L1-S1 levels were selected. Since the intraoperative EMG monitoring system was available in our hospital from April 1st, 2018, these patients were divided into two groups according to intraoperative EMG monitoring utility: (1) T-EMG group, patients in whom intraoperative T-EMG was utilized (after April 1st, 2018), and (2) non-T-EMG group, patients without T-EMG (before April 1st, 2018). Patient demographics, indications for surgery, surgical information, operative level, and pedicle screw position on postoperative CT scans were reviewed. The revision cases were recorded.

### The technique of pedicle screw placement

Pedicle diameter and length were measured preoperative on CT scans of all patients to evaluate the proper screw size. The patients were placed in the prone position on a radiolucent operating table. After completing subperiosteal dissection, the pedicle entry point was identified at the junction of the transverse process with the superior articular process of each vertebra. After the starting point was identified, an awl was used to access each pedicle, followed by a pedicle probe. After confirming four walls and a floor of the pedicle with a ball-tip sound probe, the pedicle trajectory was tapped with an undersized tap. Titanium alloy pedicle screws (stryker, US) were placed bilaterally in a standard fashion. Intra-operative anteroposterior and lateral images were obtained in both groups to confirm the pedicle screw positions. The intra-operative tangential view of the medial and lateral pedicle walls was obtained to confirm the breach of the pedicle wall if necessary.

### Pedicle screw testing

**Fig. 1:**
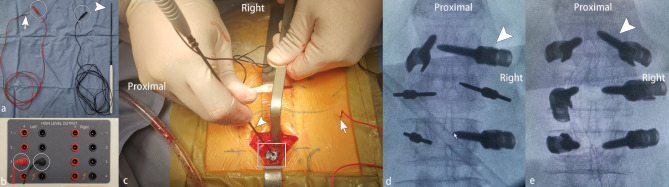
a and b, the pedicle screw test tool used intraoperatively to detect the pedicle screw (a, electrodes; b, stimulation box; arrowhead, cathode; arrow, anode; white circle, electrode plugs attached to stimulation box); c, intraoperative application of T-EMG, the cathode(arrowhead) contacted with the screw (white box), subdermal anode needle electrode inserted into the skin (arrow). d, the intra-operative anteroposterior (AP) plain of lumbar screw image and T-EMG test showed the right pedicle screw of L1 with a high possibility of internal pedicle wall breach (white arrowhead); e, the AP plain of screw position after re-insertion with T-EMG threshold > 15 mA (white arrowhead)

In the T-EMG group, each pedicle screw was individually tested by an experienced neurophysiologist. The triggering EMG technique was based on the initial report by Calancie et al [34]. Stimulation was performed using EMG with a monopolar electrode (Fig. 1a, b and c, arrowhead, cathode) and a subdermal needle electrode inserted into the para-vertebral musculature (Fig. 1a, b and c, arrow, anode). We used a pulse-train stimulation model instead of single-pulse stimulation [35]. Repetitive constant current stimulation consisting of four 0.2 ms square-wave pulses with a 2 ms interpulse interval that increased from 0 mA to 30 mA with a frequency of 3 Hz, was used through the inserted pedicle screw to evaluate the screw’s trajectory (Fig. 1c, d and e). The following muscle groups were used for the following levels of surgery: Iliopsoas L1, Adductor Longus L2–L4, Vastus Lateralis (quadriceps) L2–L4, Anterior Tibialis L4–L5, Bicep Femoris (hamstrings) L4–L5, and Gastrocnemius S1–S2.

### Pedicle screw revision

When the screw was stimulated at a threshold of ≤ 15 mA or had shown any breach sign of the medial pedicle wall on the intraoperative tangential view, it was identified as a suspected pedicle breach. All suspected violations identified by trigger-EMG (Fig. 1) or imaging were revised intraoperatively and immediately by removing the screw and examining the entire screw trajectory with the ball-tipped probe. The screw could be reinserted into the same tract, redirected, or not reinserted based on the integrity of the medial pedicle wall. The repositioned screw was re-checked using anteroposterior, lateral, and tangential view radiographs and T-EMG.

### Postoperative CT and evaluation of pedicle screw breach

**Fig. 2:**
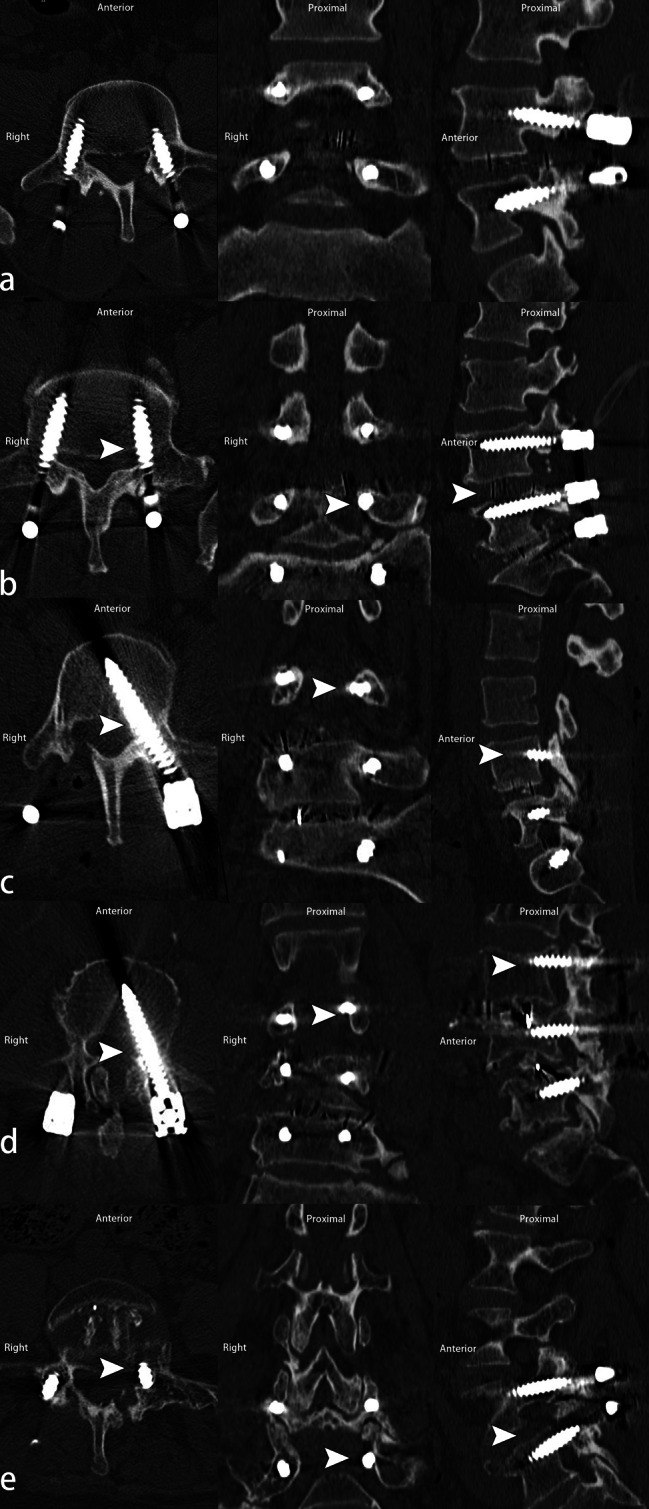
Medial/inferior screw breach definition based on horizontal, coronary, and sagittal CT scans. The image is illustrated from top to bottom as horizontal, sagittal, and coronary slices. Grade 0 (row a): screw inside the pedicle; Grade 1 (row b): perforation of the pedicle cortex by up to 2 mm; Grade 2 (row c): from 2–4 mm; Grade 3 (row d): from 4–6 mm; Grade 4(row e): by more than 6 mm. The breached screws are indexed as white arrowheads

Screw positions were evaluated one day after surgery using a high-definition CT lumbar scan (Siemens, Germany, 64-detector-row helical CT scanner). The spine protocol generated 0.5-mm source slices. The dosage parameters were 120 kV and software-based modulated mAs, with a maximum of 200 exposures. The raw data reconstructed sagittal and coronal sections of 0.5 mm thickness. According to the Laine [36] classification, pedicle screws were divided into medial/inferior and lateral/superior subgroups based on the screw breach position. The definition of the medial/inferior pedicle breach grade on CT scan based on Gertzbein-Robbins criteria [37] or Laine criteria [36] with axial, sagittal, and coronal images were shown in Fig. 2. The medial/inferior screw breach grade was staged as a screw inside the pedicle (grade 0), perforation of the pedicle cortex by up to 2 mm (grade 1), 2–4 mm (grade 2), 4–6 mm (grade 3), or > 6 mm (grade 4). According to the Gertzbein [37] and Yu [38] reports, we divided the subgroup of medial/inferior breaches into minor breaches (grades 1 and 2, breaches ≤ 4 mm) and major breaches (grades 3 and 4, breaches > 4 mm). The two groups were further divided into subgroups based on screw position (lateral/superior, medial/inferior) and breach degree (minor and major breaches). Axial, sagittal, and coronal images were independently evaluated by two spine fellow surgeons to whom the patient’s information was blind. A senior attending surgeon was consulted if there were inconsistencies. The intraobserver and interobserver reliability of three observers were analyzed by Cohen’s kappa test.

### Second revision surgery

Patient who suffered persistent radiated pain or neurological deterioration after surgery was first evaluated by X ray, CT, MRI, and inflammatory markers to clarify the exact reasons. Initially, patient was treated by conservative treatment if there were malposition of the pedicle screws without other reasons such as a nerve root compression by migration of the cage or grafted bone, haematoma formation, infection. A second surgery to revise the breached screw without T-EMG monitoring was performed when conservative treatment was failure. Before removing the violated screw, the breached medial pedicle wall and the adjacent nerve root were clearly exposed, the nerve root was carefully retracted to central canal to avoid being curled by backing rotated screw. After re-inserting the screw, in addition to the use of intraoperative fluoroscopy, the medial pedicle wall and the adjacent nerve root were also confirmed again to avoid directly contact. The postoperative CT scan after the second surgery was obtained.

### Statistics

Statistical analysis was performed using the SPSS software (version 26.0) for Windows (SPSS Inc., Chicago, IL, USA). The Student’s t-test for continuous variables which matched a normal distribution, chi-squared test, and Fisher’s exact test for categorical variables were used with a P value < 0.05 as statistically significant. All the tests were two-tailed. The intraobserver and interobserver reliability were assessed by Cohen’s kappa test, with the values ≤ 0 as indicating no agreement and 0.01–0.20 as none to slight, 0.21–0.40 as fair, 0.41– 0.60 as moderate, 0.61–0.80 as substantial, and 0.81–1.00 as almost perfect agreement [39].

## Results

A total of 713 patients with 3403 screws from L1-S1 on postoperative CT scans were included in the study. There were 327 men and 386 women, with a mean age of 60.51 years (Table 1). There were 374 cases in the T-EMG group (1723 screws, 868 vertebras, mean age 60.82 years, 177 men, 197 women) and 339 cases in non-T-EMG group (1680 screws, 845 vertebras, average age 60.17 years, 150 men, 189 women). No significant difference was observed in the disease categories, mean pedicle height and with diameter, operative time, and intraoperative blood loss between the groups (Table 1; t test).

**Table 1:** The patient demographics of T-EMG and non-T-EMG groups in our study

Group		T-EMG	Non-T-EMG	P value
Patient number		374	339	-
Screw number		1723	1680	-
Mean age		60.82	60.17	0.478
Gender	Male	177	150	0.410
	Female	197	189	
Disease	Disc herniation	166	133	0.411
	Stenosis	120	106	
	Spondylolisthesis	50	63	
	Vertebral fracture	26	19	
	Scoliosis	8	12	
	Infection	4	5	
	Tumor	0	1	
Mean pedicle diameter	Height(mm)	14.32	14.14	0.051
	Width(mm)	12.72	12.61	0.364
Number of fixed vertebrae		868	845	-
Operative time(min)		164.69	162.17	0.434
Intraoperative blood loss(ml)		324.78	321.45	0.662

The mean intraobserver kappa coefficients for T-EMG and non-T-EMG group were 0.86 and 0.86 respectively. The mean interobserver kappa coefficients for T-EMG and non-T-EMG group were 0.85 and 0.87 respectively. Kappa statistics showed high levels of agreement when the intraobserver and interobserver reliability were analyzed (Table 2).

**Table 2:** Kappa values of T-EMG and non-T-EMG groups for intraobserver and interobserver reliability

Observer		T-EMG		Non-T-EMG	
		Kappa value	95% CI	Kappa value	95% CI
Intraobserver	1	0.86	0.82–0.91	0.87	0.83–0.91
	2	0.85	0.81–0.90	0.85	0.81–0.89
	3	0.87	0.83–0.91	0.87	0.84-0.091
Average		0.86		0.86	
Interobserver	1 and 2	0.85	0.80–0.89	0.86	0.82–0.90
	1 and 3	0.87	0.83–0.91	0.89	o.86-0.092
	2 and 3	0.83	0.78–0.88	0.87	0.87–0.91
Average		0.85		0.87	

In the T-EMG group, there were 1723 screws, of which 134 were found to be breached, and the overall screw breach rate was 7.78% (134/1723). There were 1680 screws in the non-T-EMG group, with 189 screws penetrating the pedicle wall. The overall screw breach rate was 11.25% (189/1680). There was a significant difference in the overall screw breach rate between the 2 groups (Table 3; χ²= 11.942, p = 0.001).

**Table 3:** The total number of breaches and non-breach screws in T-EMG and non-T-EMG groups

Breach grade	T-EMG	Non-T-EMG	P value
Screw breach	134(7.78%)	189(11.25%)	0.001
No breach	1589(92.2%)	1491(88.75%)	
Total	1723(100%)	1680(100%)	

On the basis of the position subgroups, 108 of 134 (80.60%) screws violated the medial/inferior cortex (101 cases of grade 1, 6 of grade 2, and 1 of grade 3), and 26 (19.40%) breached the lateral/superior pedicle cortex in the T-EMG group. The number of screw breaches per total number of screws in each segment is presented in Table 4. In the non-T-EMG group, 150/189 (79.37%) screws penetrated medially/inferiorly (123 of grade 1, 17 of grade 2, 5 of grade 3, and 5 of grade 4) and 39 (20.63%) penetrated laterally/superiorly. The number of screw breaches per total number of screws in each segment is presented in Table 5. There was a statistically significant difference in the medial/inferior breach rate between the T-EMG group (6.27%, 108/1723) and non-T-EMG group (8.93%, 150/1680) (χ²= 12.014, p = 0.002). Although there was a trend of difference between the groups (T-EMG, 1.51% (26/1723) vs. non-T-EMG, 2.32% (39/1680)) in terms of the lateral/superior breach screws, the difference was not statistically significant (Table 6, χ²=3.423, p = 0.064).

The minor and major medial/inferior screw breach rates were 6.21% (107/1723) and 0.06% (1/1723) in the T-EMG group, and 8.33% (140/1680) and 0.60% (10/1680) in the non-T-EMG group, respectively. There were significant differences in major and minor medial/inferior screw breach rates in the T-EMG and non-T-EMG groups (Table 6, χ²=16.950, p = 0.001).

**Table 4:** The number of screw breaches per total number of screws in each segment in the T-EMG group

Breach grade	L1	L2	L3	L4	L5	S1	Total
Grade 0	52(3.27%)	79(4.97%)	156(9.82%)	499(31.40%)	592(37.26%)	211(13.28)	1589(100%)
Medial/inferior breach	9(8.33%)	4(3.70%)	14(12.96%)	32(29.63%)	36(33.33%)	13(12.04%)	108(100%)
Grade 1	8(7.92%)	4(3.96%)	11(10.89%)	31(30.69%)	35(34.65%)	12(11.88%)	101(100%)
Grade 2	1(16.67%)	0(0%)	3(50.00%)	1(16.67%)	0(0%)	1(16.67%)	6(100%)
Grade 3	0	0	0	0	1(100%)	0	1(100%)
Grade 4	0	0	0	0	0	0	0
Lateral/superior breach	11(42.31%)	3(11.54%)	1(3.85%)	9(34.62%)	2(7.69%)	0	26(100%)
Total	72	86	171	540	630	224	1723

**Table 5:** The number of screw breaches per total number of screws in each segment in the non-T-EMG group

Breach grade	L1	L2	L3	L4	L5	S1	Total
Grade 0	37(2.48%)	64(4.29%)	162(10.87%)	450(30.18%)	562(37.69%)	216(14.49)	1491(100%)
Medial/inferior breach	12(8.00%)	4(2.67%)	26(17.33%)	44(29.33%)	44(29.33%)	20(13.33%)	150(100%)
Grade 1	12(9.76%)	3(2.44%)	20(16.26%)	34(27.64%)	39(31.71%)	15(12.20%)	123(100%)
Grade 2	0(0%)	0(0%)	5(29.41%)	8(47.06%)	1(5.88%)	3(17.65%)	17(100%)
Grade 3	0	1(20%)	1(20%)	1(20%)	2(40%)	0	5(100%)
Grade 4	0	0	0	1(20%)	2(40%)	2(40%)	5(100%)
Lateral/superior breach	1(2.56%)	8(20.51%)	4(10.26%)	18(46.15%)	8(20.51%)	0	39(100%)
Total	50	76	192	512	614	236	1680

**Table 6:** Comparison of the subgroup of screw breach rates

Breach grade		T-EMG	Non-T-EMG	P value
Lateral/superior breach		26(1.5%)	39(2.3%)	0.064
Medial/inferior breach		108(6.3%)	150(8.9%)	0.002
Medial/inferior breach	Grade 1 + 2	107(6.2%)	140(8.3%)	0.001
	Grade 3 + 4	1(0.1%)	10(0.6%)	0.001
No breach		1589(92.2%)	1491(88.8%)	
Total		1723(100%)	1680(100%)	

**Fig. 3:**
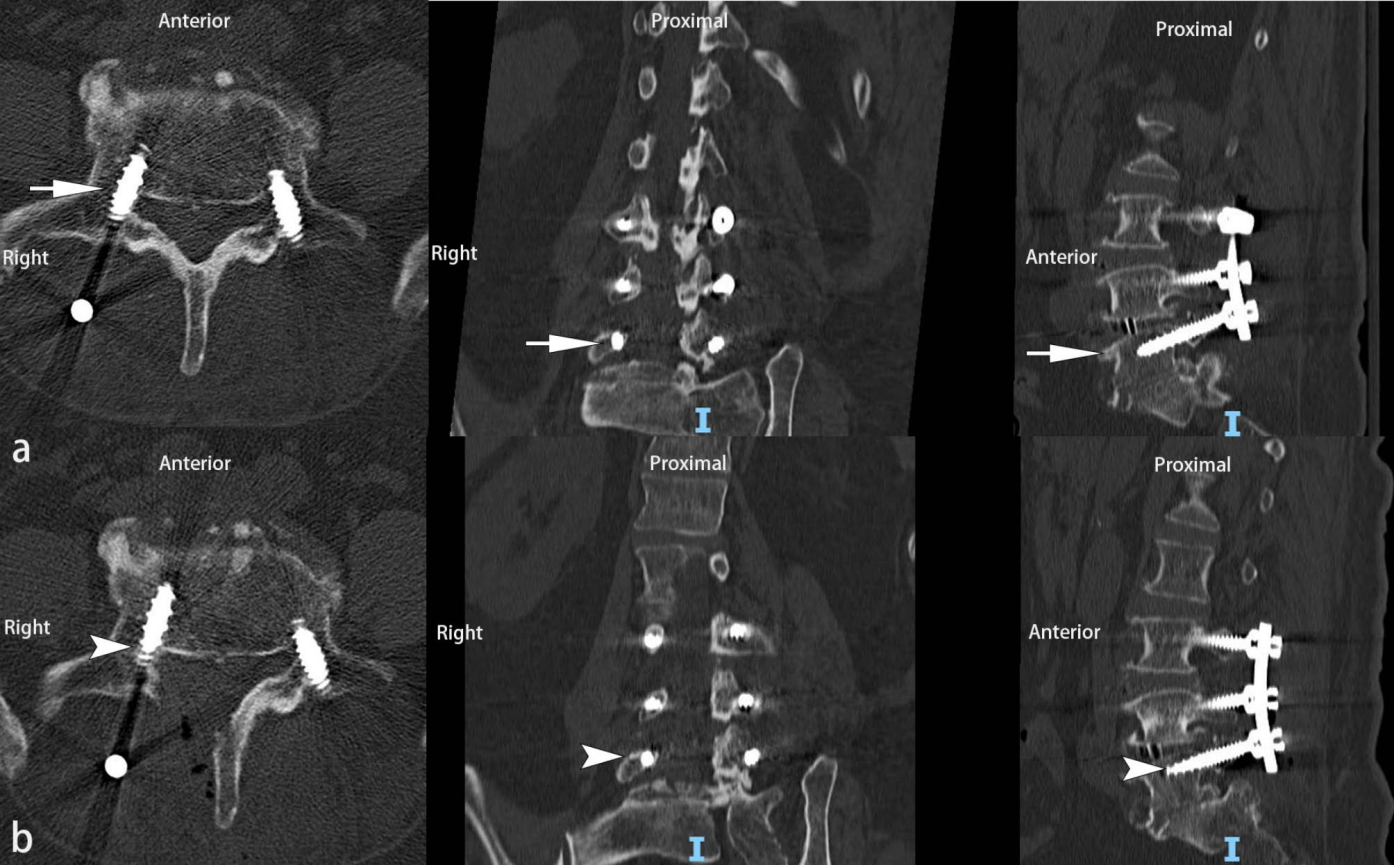
A 71-year-old woman underwent transforaminal lumbar interbody fusion (TLIF) from L3-L5. At postoperative day 1, the patient suffered persistent radiated pain from the back to the right foot. The postoperative CT scan showed that a minor medial screw breach (< 2 mm, grade 1) of the right L5 pedicle (a, white arrow). The patient received two weeks of conservative treatment without relief. The patient received a second surgery to revise the breached screw of L5 on the third postoperative week without T-EMG monitoring. Before removing the violated screw, the breached medial pedicle wall and the adjacent nerve root were clearly exposed, the nerve root was carefully retracted to central canal to avoid being curled by backing rotated screw. After re-inserting the screw, in addition to the use of the intraoperative X ray, the medial pedicle wall and the adjacent nerve root were confirmed again to avoid directly contact. The radiated pain was entirely resolved after the revision surgery. The CT scan showed a revised screw without the pedicle wall breach (b, white arrowhead)

**Fig. 4:**
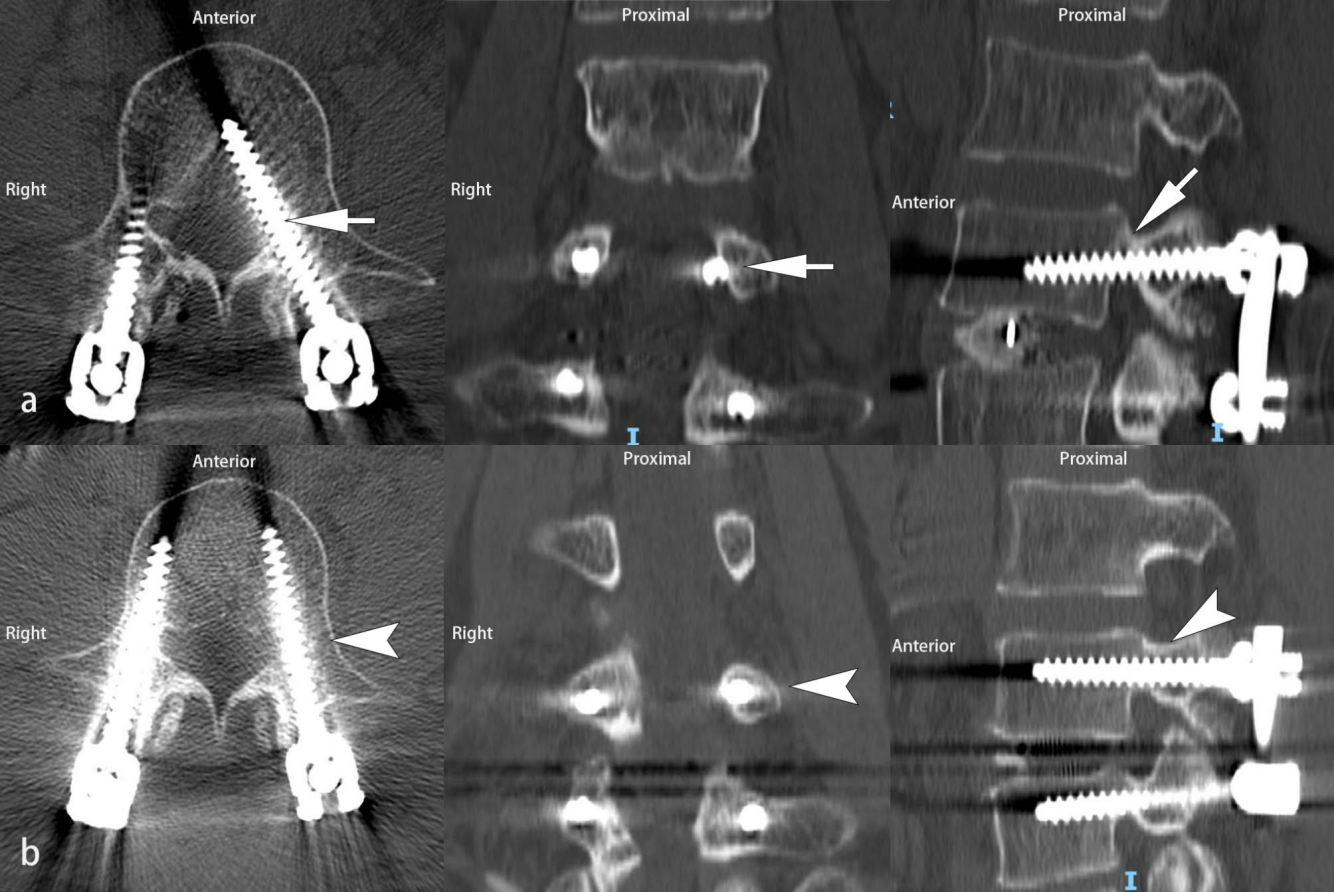
A 65-year-old woman undergoing L4-5 TLIF had severe radiated pain of the left lower extremity with weak muscle strength at postoperative day 1. The postoperative CT scan revealed a major pedicle screw breach (> 6 mm, grade 4) of the left L4 pedicle (a white arrow). Revision surgery was scheduled immediately. The postoperative CT scan showed that the revised screw was inside the pedicle (b, white arrowhead). The patient recovered without consequence

Six screws (6 patients) underwent revision due to persistent postoperative neurological deficits, all of which were in the non-T-EMG group. There was one for grade 1 (Fig. 3), one for grade 2, one for grade 3, and three for grade 4 (Fig. 4). No revision screws were used in the T-EMG group. There was a statistically significant difference in the revision rate between patients with screw breach in the T-EMG group (0%, 0/134) and those in the non-T-EMG group (3.17%, 6/189) (Table 7, Fisher’s exact test, p = 0.044). When comparing the overall revision rates of minor and major screw penetration, the minor screw breach revision rate (0.81%, 2/247) was significantly lower than that of major screw breaches (36.36%, 4/11) (Table 8, Fisher exact test, p = 0.001).

**Table 7:** Revision rate of the breached screw in T-EMG and non-T-EMG groups

	T-EMG	Non-T-EMG	P values
No revised breach	134(100%)	183(96.83%)	0.044
Revised breach	0	6(3.17%)	
Total	134(100%)	189(100%)	

**Table 8:** Comparison of the total revision rate of major and minor screw breaches

	Non-revision	Revision	P value
Minor breaches	245(97.2%)	2(33.33%)	0.001
Major breaches	7(2.78%)	4(66.67%)	
Total	252(100%)	6(100%)	

## Discussion

Pedicle screw breach in posterior lumbar surgery is common [36] and severe pedicle screw breach can lead to persistent neurological deficit [10, 40]. There are many ways to avoid pedicle screw breach, such as an intraoperative pedicle ball-tip probe [14], intraoperative X-ray [41], O-arm [42]or three-dimensional CT [43], 3D printed screw guide templates [20], robot-assisted pedicle screw placement [22], the probe with electronic conductivity device [23, 24]. Triggered EMG as a method for evaluating pedicle screw placement accuracy was first reported by Calancie in 1994 [34]. However, its accuracy in reducing pedicle screw breach rates remains controversial [27, 31–33]. Most studies reported the sensitivity and specificity of screw placement accuracy under different T-EMG thresholds [26, 29, 44], and there are few reports comparing the pedicle screw breach rate with or without the application of T-EMG directly [45, 46]. This is the first study to directly compare the pedicle screw breach rate and reoperation rate in posterior spine pedicle screw fixation with or without intraoperative T-EMG monitoring based on the postoperative CT scan.

Laine et al. [36] classified pedicle screw breaches into medial, lateral, superior, and inferior according to the penetration position. Due to the anatomical relationship between the pedicle and the relevant nerve root, medial or inferior pedicle breach is at high risk for nerve injury [10], while the lateral or superior breach is at low risk [9, 37]. We divided pedicle screw breach into medial/inferior and lateral/superior subgroups. Gertzbein et al. [37] reported that neurological deficits rarely occurred when the pedicle screw breach was less than 4 mm (grade 1–2). The probability increased dramatically when the breach was greater than 4 mm (grade 3–4). Therefore, we staged pedicle screw breaches as minor breaches (≤ 4 mm, grade 1–2) and major breaches (> 4 mm, grade 3–4).

### The pedicle screw breach rate of the T-EMG vs. Non-T-EMG group

In the present study, the overall pedicle screw breach rate in the non-T-EMG group (11.25%) was significantly higher than that of the T-EMG group (7.78%). When comparing by the subgroup, the medial/inferior pedicle penetration rate between T-EMG (6.27%) and non-T-EMG (8.93%) groups significantly differed. In contrast, the lateral/superior pedicle subgroup showed no significant differences (T-EMG, 1.51% VS non-T-EMG 2.32%, p > 0.05). The results suggest that applying intraoperative T-EMG during posterior screw placement could effectively reduce the overall breach rate of the pedicle screw, especially for medial/inferior breaches. However, it did not reduce the rate of lateral/superior screw breaches. This was due to the anatomical relationship between the pedicle and relevant nerve root. The exit nerve root typically runs beside the medial or inferior wall. The screw-nerve distance was very close when the pedicle screw penetrated the medial/inferior pedicle wall. It is much easier for T-EMG monitoring to trigger a positive reaction [47]. The current impedance is extremely high for lateral/superior pedicle screw breach because the screw-nerve distance is too long; the 30mA threshold is not sufficient to trigger a positive T-EMG reaction [34, 46].

### The revision rate was related to breach grade and screw-nerve root distance

Perumal et al. [45] compared postoperative revision rates in patients with and without T-EMG. One of 296 patients in the T-EMG group underwent revision, but 6 of the 222 patients underwent revisions in the non-T-EMG group. Tani et al [48] reported a 3.3% (51/1536) medial or inferior pedicle wall breach rate without neurological complications when using T-EMG alone as an intra-operative pedicle trace-monitoring tool. In our study, six patients (six screws) underwent revision surgeries for patients in the non-t-EGM group, including two cases of minor breaches and four cases of major breaches. Surprisingly, none of the patients in the T-EMG group underwent screw revision surgery. There was a statistically significant difference between the two groups (T-EMG, 0% vs. non-T-EMG, 3.17%), suggesting that intraoperative T-EMG could effectively reduce the breached screw revision rate.

studies have reported that medial/inferior breaches exceeding 4 mm usually induce symptomatic neurological deficit [10, 11, 37]. Our study similarly concluded that grade 1 and 2 medial/inferior breaches had a low revision rate (0.81%, 2/247), and grade 3 and 4 breaches increased the revision rate dramatically (36.36%, 4/11). We found two interesting results in our study. First, the major breaches with neurological deficits requiring revision were all in the non-T-EMG group (four screws). Second, regardless of whether T-EMG was used, some patients with major breaches(seven screws)had no clinical symptoms. It has also been reported that a major screw breach did not necessarily cause clinical symptoms [10, 40, 41], suggesting that the degree of screw breach could not simply be used to decide whether revision was needed.

Based on our experience, we speculate that the screw-nerve root distance determines whether a breach causes a neurological deficit. Theoretically, a breach screw should contact the relevant nerve root to cause clinical symptoms. The possibility of screw-related neurological deficits is low if the screw is at a long distance from the exit nerve root. Montes et al. [49] discovered that the integrity of the medial wall of the pedicle screw did not affect the threshold of T-EMG when the distance between the nerve root and screw was greater than 8 mm. Skinner et al. [47] reported that the distance between the screw and nerve root significantly changes the T-EMG threshold. For the breached medial pedicle wall, the threshold stimulation of the nearby root is more a function of Coulomb’s law than Ohm’s law. The current flow escaping from the breached pedicle was attenuated by the square of the distance from the excitable tissue. Conversely, the threshold stimulation is significantly decreased if the pedicle screw is in contact with the nerve root. De Blas et al. [50] and Montes et al. [49] reported in animal experiments that the contact between the breach screw and the exit nerve root beside the medial/inferior breach pedicle wall could significantly decrease the threshold. These conclusions are in accordance with our data. There were false-negative cases (screw breach with ≥15 mA threshold) with a major or minor breach in the T-EMG group, probably due to the long distance between the screw-nerve root.

Not surprisingly, patients with major breaches were at a high risk for revision in the non-T-EMG group. Interestingly, although most of the minor breaches were asymptomatic (99.19%, 245/247), two patients in the non-T-EMG group required screw revision (two cases, Fig. 2). The reason might be the tight contact between the breach screw and nerve root. In the non-T-EMG group, symptomatic breach could not be identified by intra-operative radiography without T-EMG. There were no revision cases in the T-EMG group, mainly because the T-EMG monitoring could identify the breach, whether major or minor, due to the relatively low threshold stimulation when there was a tight contact between the nerve and the breach screw; therefore, revision was performed in a timely manner during surgery. Malham et al. [46], Soriano [41], and Duffy [51] reported that no patient needed revision despite the breach of the screw when intraoperative T-EMG monitoring was applied. This again supports the hypothesis that the screw-nerve root distance determines whether breaches cause clinical symptoms.

### Stimulation threshold of T-EMG

Screw placement accuracy in our study was 92.2% (1589/1723). Several studies have found that the accuracy of screw placement cannot reach 100% using 5-15mA [28, 44, 51–55] as the stimulation threshold. The false-negative result of T-EMG might be related to factors other than the screw-nerve root distance: 1). Anatomical factors include different thicknesses of cortical bone [32, 33], abnormal bone structure [32], abnormal muscle innervation [14], and chronic nerve root compression before surgery [32, 33, 56]; 2). Technical factors, screw diameter [57], screw structure [58], material composition of the screw [59], coated screw [60], the way pedicle screw measured [60], and strength-duration time constant [47]; 3). Surgical factors include muscle relaxants [61, 62], pedicle screw distance from nerve [63], and excess fluid in the surgical field [57]. Therefore, T-EMG should be combined with an intraoperative X-ray and ball-tip probe to minimize the breach of the pedicle screw [32].

There is no consensus on the screw stimulation threshold during T-EMG monitoring. A recently published meta-analysis suggested that the stimulation threshold at 8mA had higher specificity and sensitivity, while increasing the threshold resulted in higher specificity but not sensitivity [64]. We selected 15mA as the stimulation threshold for the following reasons. First, the T-EMG threshold reported in the literature ranged from 5-15mA [28, 29, 44, 51–55, 63]; however, none of the thresholds obtained 100% accuracy. 15mA is the upper limit of the threshold reported in the literature. Using a threshold of 15mA, the probability of screw breach was low [53, 63]. Therefore, 15mA is a more conservative threshold and is used as the recommended threshold [47]. Second, we included many patients with chronic degenerative spinal disease. For patients with chronic nerve root compression, the threshold of T-EMG would significantly increase [32, 33, 56]. Thus, the application of a slightly higher threshold such as 15 mA, is more suitable [28]. Third, screws with titanium alloy composition are less conductive than stainless steel screws, which will significantly increase the threshold of T-EMG; a lower threshold might result in negative results [65].

### Limitations

Our study had several limitations. First, this is a retrospective study, and the groups were assigned based on the availability of a neuromonitoring device. Therefore, there are huge biases to select the patient cases based on the surgeons’ preference and learning curve as well as the difficulty and type of surgery. Second, the diseases were from a broad category, and the patient cohort contained different diseases with various bone qualities, anatomy, and pedicle diameter, which might complicate the comparison difference. However, most the patients had degenerative diseases, and the disease difference between the two groups was insignificant, the pedicle diameters of two groups were similar, which could minimize bias. Third, surgeries were performed at a long-time frame (non-T-EMG group and T-EMG group were performed before and after April 2018, respectively), surgeon’s experience was increasing during the past years, which bias the results. However, the spinal surgeries were performed by a senior attending physicians who was well trained with rich experience, which could reduce this bias.

## Conclusion

The T-EMG technique has good clinical utility for evaluating pedicle screw accuracy. Compared to intraoperative fluoroscopy alone, it is of great value in improving the accuracy of screw placement and reducing the rate of symptomatic pedicle screw breach and the related pedicle screw revision. The screw-nerve root distance plays a vital role in causing symptomatic pedicle screw breaches. T-EMG can be used as a standard tool for assessing the accuracy of pedicle screw placement to reduce incorrect screw placement.

## Data Availability

The data presented in this study are available from the corresponding author.

## List of Abbreviations

EMG: Electromyography
T-EMG: Triggered electromyographic
CT: Computed tomography
